# Matching-adjusted indirect comparison of efficacy and safety of lisocabtagene maraleucel and mosunetuzumab for the treatment of third-line or later relapsed or refractory follicular lymphoma

**DOI:** 10.1186/s40164-025-00610-1

**Published:** 2025-03-05

**Authors:** Loretta J. Nastoupil, Ashley Bonner, Pearl Wang, Lamees Almuallem, Jigar Desai, Thalia Farazi, Jinender Kumar, Saurabh Dahiya

**Affiliations:** 1 Southwest Oncology, CommonSpirit Mercy, 1 Mercado St, STE 100, Durango, CO USA; 2 EVERSANA, Burlington, ON Canada; 3 Bristol Myers Squibb, Princeton, USA; 4 Bristol Myers Squibb, San Francisco, USA; 5 Stanford University School of Medicine, Stanford, USA; 6 University of Maryland Greenebaum Comprehensive Cancer Center, Baltimore, USA

**Keywords:** CAR T-cell therapy, Lisocabtagene maraleucel, Mosunetuzumab, Indirect treatment comparison, Matching-adjusted indirect comparison, CD20 × CD3 bispecific T-cell–engaging monoclonal antibodies

## Abstract

**Background:**

The treatment landscape for relapsed or refractory (R/R) follicular lymphoma (FL) has changed with the introduction of anti-CD19 chimeric antigen receptor T-cell therapies, including lisocabtagene maraleucel (liso-cel) and CD20 × CD3 bispecific T-cell–engaging monoclonal antibodies such as mosunetuzumab. Liso-cel and mosunetuzumab have demonstrated positive benefit-risk profiles for third-line or later (3L+) treatment of patients with R/R FL and are approved treatments for these patients. In the absence of a prospective, randomized study, we conducted an unanchored matching-adjusted indirect comparison (MAIC) to assess the efficacy and safety of liso-cel and mosunetuzumab for 3L+ treatment in patients with R/R FL.

**Methods:**

Unanchored MAICs were performed to estimate relative treatment effects between TRANSCEND FL (NCT04245839) and GO29781 (NCT02500407). For TRANSCEND FL, the leukapheresis set (*N* = 114) was used for primary comparisons of the following efficacy endpoints: objective response rate (ORR), complete response (CR) rate, duration of response (DOR), and progression-free survival (PFS). The treated set (*N* = 107) was used for comparisons of the following safety endpoints: cytokine release syndrome (CRS), neurological events (NE), serious infections, and use of corticosteroids or tocilizumab for CRS. Sensitivity analyses were conducted for efficacy using the TRANSCEND FL treated efficacy set (*N* = 101).

**Results:**

After adjustment, liso-cel was associated with higher ORR (odds ratio [OR] = 3.78, 95% confidence interval [CI] 1.48‒9.67]) and CR rate (OR = 6.46, 95% CI 2.85‒14.65), and improved DOR (hazard ratio [HR] = 0.45, 95% CI 0.26‒0.77) and PFS (HR = 0.28, 95% CI 0.16‒0.49) compared with mosunetuzumab. Results remained consistent across sensitivity analyses. Liso-cel had a lower incidence of grade ≥ 3 CRS (OR = 0.45, 95% CI 0.04‒5.13), grade 3‒4 serious infections (OR = 0.35, 95% CI 0.12‒1.03), and corticosteroid use for CRS management (OR = 0.14, 95% CI 0.03‒0.65); however, liso-cel exhibited higher incidence of any-grade CRS (OR = 1.86, 95% CI 1.01‒3.43), any-grade NEs (OR = 2.16, 95% CI 0.72‒6.44), and tocilizumab use for CRS management (OR = 2.27, 95% CI 0.86‒5.99).

**Conclusions:**

Findings highlight a potential positive benefit-risk profile of liso-cel over mosunetuzumab as a 3L+ treatment for R/R FL.

**Supplementary Information:**

The online version contains supplementary material available at 10.1186/s40164-025-00610-1.

## Background

Follicular lymphoma (FL) is the most common type of indolent non-Hodgkin lymphoma (NHL) [1], representing about 35% of all NHL cases and 70% of indolent lymphomas diagnosed in the United States and Western Europe [1–3]. FL predominantly affects adults over the age of 50, with slightly higher rates found in female patients in Europe and the United Kingdom [4]. As more than 90% of patients present with advanced-stage disease at diagnosis, early-stage FL is rare [1]. The introduction of rituximab in 1998 marked a pivotal shift in the management of FL, significantly improving overall survival (OS) in the FL population, with approximately 80% of patients experiencing 10-year OS [1]. However, FL remains a disease in which most patients will experience multiple relapses, with existing treatments primarily focused on alleviating symptom burden, and prolonging survival [1, 5].

First- and second-line (2L) treatment options for FL typically include an anti-CD20 monoclonal antibody, either independently or in combination with chemotherapy [6]. Although autologous or allogeneic hematopoietic stem cell transplantation (HSCT) may be considered for 2L therapy in a select population (i.e., mainly young and fit), its use is limited in clinical practice because of increased risk of developing secondary malignancies and because of the potential impacts of toxicity on quality of life [7–9]. Notably, progression of disease ≤ 24 months (POD24) is observed in approximately 20% of patients who receive first-line chemoimmunotherapy [5, 10]. Moreover, POD24 appears to be correlated with prognosis [5, 10]. Specifically, patients with relapsed or refractory (R/R) FL meeting POD24 criteria have a 5-year OS of 50%, versus 90% in patients without early progression [5].

For patients with R/R FL, there is currently no established standard of care (SOC) after at least two lines of therapy (LOT) [11, 12]. Conventional chemoimmunotherapy regimens, alongside experimental agents and combination therapies such as lenalidomide with rituximab, are commonly used. Axicabtagene ciloleucel (axi-cel) and tisagenlecleucel (tisa-cel) are anti-CD19 chimeric antigen receptor (CAR) T-cell therapies that were approved by the United States Food and Drug Administration (FDA) for the treatment for R/R FL after two or more LOTs in 2021 and 2022, respectively [13, 14], along with the CD20 × CD3 bispecific T-cell–engaging monoclonal antibody mosunetuzumab in 2022 [15, 16]. Collectively, these therapies provided new treatment options for patients with advanced R/R FL. Nevertheless, the prognosis of R/R FL after at least two LOTs remains poor [17, 18]. The approvals of axi-cel and tisa-cel were based on the phase 2 study results of ZUMA-5 (NCT03105336) and ELARA (NCT03568461), respectively [13, 14, 19, 20]. Both axi-cel and tisa-cel have demonstrated high ORR and CR rates in patients with R/R FL [19].

More recently, lisocabtagene maraleucel (liso-cel), an autologous, CD19-directed, 4-1BB CAR T-cell product, was approved by the FDA for the third-line or later (3L+) treatment of R/R FL [21]. The efficacy and safety of liso-cel in adult patients with 2L or later R/R FL and marginal zone lymphoma were evaluated in TRANSCEND FL (NCT04245839), a phase 2, single-arm, open-label, multicohort, multicenter study [22]. In this study, patients with 3L+ R/R FL treated with liso-cel demonstrated an improved objective response rate (ORR) of 97%, with 94% achieving a complete response (CR). Responses were durable, with the median duration of response (DOR) not reached (NR) at a median follow-up of 16.6 months, with 81.9% of patients achieving 12-month DOR [22]. Similarly, progression-free survival (PFS) was NR at a median follow-up of 17.5 months, with 80.7% of patients achieving 12-month PFS [22]. Liso-cel also demonstrated a manageable safety profile, with low incidences of severe cytokine release syndrome (CRS) and neurological events (NE) [22].

Similarly, the phase 2, single-arm, multicenter GO29781 study (NCT02500407) evaluated the safety and antitumor activity of mosunetuzumab in patients with 3L+ R/R FL, demonstrating efficacy with high response rates (ORR of 80% and CR rate of 60%) and sustained remission after receiving at least two LOTs, including an anti-CD20 monoclonal antibody and an alkylating agent [15]. Patients achieving CR rate after eight cycles completed treatment without further cycles, while those with partial response (PR) or stable disease underwent a total of 17 cycles [15]. Mosunetuzumab treatment was also associated with adverse events (AE), including CRS and NEs [15]. Indirect treatment comparison (ITC) methods have been performed to assess the safety and efficacy of the CAR T-cell therapies axi-cel and tisa-cel compared with mosunetuzumab in the treatment of patients with R/R FL after at least two LOTs [23, 24]. However, no comparison of liso-cel and mosunetuzumab has yet been conducted.

Due to the absence of prospective, randomized data comparing these emerging therapies, the differences in efficacy and safety between treatment options currently remain unclear. In the absence of such comparisons, ITCs can be used to inform comparisons between trials [25]. Matching-adjusted indirect comparison (MAIC) is a form of ITC that compares treatment effects while adjusting for confounding bias arising from study differences, such as observed differences in eligibility criteria and patient baseline characteristics [25, 26]. The objective of this study was to compare the efficacy and safety of liso-cel with mosunetuzumab in the treatment of patients with R/R FL, utilizing an unanchored MAIC of individual patient data (IPD) from the TRANSCEND FL study and published summary-level data from the GO29781 study.

## Methods

### Data sources

The primary data source for liso-cel was IPD from the TRANSCEND FL study (data cutoff: January 27, 2023; median [interquartile range (IQR)] follow-up: 19.25 months [15.08‒21.52]). The primary endpoint was ORR, and secondary endpoints were CR rate, DOR, PFS, OS, and safety outcomes.

A systematic literature review (SLR) was conducted to identify evidence on the clinical efficacy and safety of approved as well as investigational therapies for the treatment of adult patients with R/R FL (grade 1‒3a) in the 3L+ population [27]. The SLR searched studies published between January 1, 1998, and September 17, 2022, using Ovid MEDLINE^®^, Embase, and Cochrane Central Register of Controlled Trials, with supplemental hand searches of key conferences and bibliographies of recent relevant SLRs. Results from the SLR identified GO29781 as the key source of efficacy and safety data for mosunetuzumab in the 3L+ R/R FL population [15]. GO29781 (NCT02500407) was a global, phase 2, single-arm, multicenter study that evaluated the efficacy and safety of mosunetuzumab in patients with R/R FL. The primary endpoint for the GO29781 study was independent review committee (IRC)–assessed CR rate in all enrolled patients, while key secondary endpoints were investigator-assessed CR rate, IRC- and investigator-assessed ORR, DOR, PFS, OS, and AEs. The enrolled intention-to-treat (ITT) population (*N* = 90) from GO29781 (data cutoff: August 27, 2021; median [IQR] follow-up: 18.3 months [13.8‒23.3]) was utilized to assess the efficacy and safety endpoints of mosunetuzumab.

### Study comparison

To ensure alignment between the efficacy and safety sets in both studies, the TRANSCEND FL leukapheresis set (ITT; *n* = 114), defined as enrolled participants who underwent leukapheresis, was used for liso-cel efficacy outcomes. The GO29781 enrolled set (*n* = 90), defined as participants who received the initial treatment (mosunetuzumab), was used for mosunetuzumab efficacy outcomes. For safety outcomes, the TRANSCEND FL treated set (*n* = 107), defined as participants who received a conforming dose of liso-cel, was used. For mosunetuzumab, the GO29781 treated set (*n* = 90), defined as the ITT population who enrolled in the GO29781 study and received the initial treatment (mosunetuzumab), was used. Table 1 provides a summary of the data sets and outcomes used for TRANSCEND FL and GO29781.

**Table 1:** Summary of datasets and outcomes for TRANSCEND FL and GO29781

Study name	Treatment arm	Data cutoff	Median study follow-up, months (IQR)	Analysis set	*N*	Outcomes
**Efficacy outcomes**						
TRANSCEND FL^a^	Liso-cel	Jan 27, 2023^b^	19.25 (15.08‒21.52)	Leukapheresis set (ITT)	114	• ORR • CR rate • PFS • DOR • OS^c^
GO29781^b^	Mosunetuzumab	Aug 27, 2021^b^	18.3 (13.8‒23.3)	Enrolled set (ITT)	90	
**Safety outcomes**						
TRANSCEND FL^a^	Liso-cel	Jan 27, 2023^b^	19.25 (15.08‒21.52)	Treated set	107	• CRS (any grade and grade ≥ 3) • NE (any grade and grade ≥ 3) • Grade 3‒4 serious infections • Use of corticosteroid and tocilizumab for CRS management
GO29781^b^	Mosunetuzumab	Aug 27, 2021^b^	18.3 (13.8‒23.3)	Treated set	90	

^a^Per individual patient-level data. ^b^Per primary publication by Budde et al. 2022 [15]. ^c^Due to differences in follow-up times and a low number of events, OS was not included in this MAIC

*CR* complete response, *CRS* cytokine release syndrome, *DOR* duration of response, *ITT* intention to treat, *IQR* interquartile range, *liso-cel* lisocabtagene maraleucel, *N* sample size, *NE* neurological event, *ORR* objective response rate, *OS* overall survival, *PFS* progression-free survival

Differences in baseline characteristics between the study populations were assessed using the absolute value of the standardized mean difference (SMD), a scale-invariant measure of imbalance between groups [28]. An SMD ≥ 0.1 was indicative of a potentially important imbalance between studies. For a given covariate, a reduction in the SMD after adjusting signified a reduction in imbalance [28].

### Study design

Both studies were multicenter, global, single-arm investigations of treatment options for R/R FL. Because mosunetuzumab is a bispecific T-cell engager, leukapheresis and lymphodepletion therapy are not applicable for the GO29781 study. In TRANSCEND FL, patients underwent screening, followed by enrollment and leukapheresis before liso-cel administration. The median (range) time from leukapheresis to product availability was 29 days (19‒55). 38% of patients in TRANSCEND FL received bridging therapy for disease control at the discretion of the treating clinician during the liso-cel manufacturing process; 34% received systemic therapy only and 4% received radiotherapy only. Most bridging therapies were combination therapies, primarily rituximab plus gemcitabine and oxaliplatin (corticosteroids alone were not used as a bridging therapy; full details on the specific bridging therapies used in 2L or later R/R FL are available in the supplementary appendix to the primary publication [22]). In contrast, patients enrolled in the GO29781 study received mosunetuzumab immediately after study enrollment and were treated with corticosteroids as a premedication to reduce the risk of CRS and NE side effects (TRANSCEND FL did not proactively use corticosteroids to prevent CRS and NEs) [15]. Based on this difference in study design, efficacy endpoints were compared using the leukapheresis set (ITT) from TRANSCEND FL versus the enrolled set (ITT) from GO29781. Sensitivity analyses were conducted to compare efficacy endpoints from the infusion of liso-cel (liso-cel–treated efficacy set, *n* = 101).

The eligibility criteria for the 3L+ R/R FL populations in the two studies were largely aligned with minor differences. TRANSCEND FL enrolled patients who received allogeneic HSCT and only excluded patients who received allogeneic HSCT within 90 days of leukapheresis. In contrast, GO29781 excluded all allogeneic HSCT and autologous HSCT within 100 days before the first mosunetuzumab administration.

### Patient characteristics

Of 21 baseline patient characteristics reported in both studies, differences in definitions were noted for bulky disease, refractory to last therapy, and prior LOT. Such differences were addressed by reclassifying or recalculating the corresponding variables within the TRANSCEND FL IPD to match the definitions reported in GO29781 (Additional file 1: Table S1). Definitions and categorizations for the remaining patient characteristics were already similar; therefore, no additional alignment was required.

### Definitions of outcome measures

Efficacy outcomes included ORR, CR rate, DOR, and PFS. Due to differences in median follow-up times (TRANSCEND FL, 19.25 months; GO29781, 18.3 months) and a low number of events, OS was not included in this MAIC. Median survival was NR in both studies and 12-month survival probability was 92.1% and 93.0% in 3L+ patients in TRANSCEND FL and GO29781, respectively [15, 22]. For the assessment of ORR and CR rate, both studies reported response outcomes by IRC based on positron emission tomography/computed tomography scans, and both utilized the Lugano classification [29]. For DOR and PFS, TRANSCEND FL and GO29781 used different censoring rules, as outlined in their respective study protocols and likely determined based on prior regulatory discussions. In TRANSCEND FL, the primary analysis (per IRC assessment) used the European Medicines Agency censoring rules, where the start of new antilymphoma therapy was considered an event. In contrast, the primary analysis in GO29781 for DOR and PFS (per IRC assessment) followed the FDA censoring rules, where the start of new antilymphoma therapy was censored. As a result, the censoring rules in TRANSCEND FL were rederived to align with those used in GO29781.

Safety outcomes in both studies included several AEs of interest as follows: (i) any-grade and grade ≥ 3 CRS following the Lee criteria [30], (ii) any-grade NEs, encompassing confusional state, disturbance in attention, cognitive disorder, aphasia, seizures, and encephalopathy, (iii) grade 3‒4 serious infections, and (iv) the use of corticosteroids or tocilizumab for managing CRS. Both studies had similar definitions for CRS and serious infections, while the definitions for study-specific NEs varied. In TRANSCEND FL, NEs were reported as investigator-identified neurological AEs, whereas in GO29781, NEs were assessed by the investigator based on the “MedDRA high-level group terms” search query. This discrepancy could not be adjusted using rederivation from TRANSCEND FL IPD and was acknowledged as a minor limitation in the study. Safety comparisons between NE outcomes were performed, and no outcomes were rederived from TRANSCEND FL for alignment with GO29781.

### Rank ordering of prognostic factors and treatment-effect modifiers

Study eligibility and baseline characteristics were considered as clinical factors that potentially required population adjustment. The identification of relevant clinical factors for matching and adjustment involved the following: (i) a review of clinical factors adjusted for in recently published MAICs in 3L+ FL [23, 24]; (ii) a review of clinical factors reported in the TRANSCEND FL and GO29781 studies, and (iii) input from clinical experts. A total of 21 potentially important clinical prognostic factors and treatment-effect modifiers were identified and rank-ordered by clinical experts based on their prognostic strength or degree of treatment-effect modification for the outcomes of interest (Additional file 1: Table S2). Separate factor-ranking exercises were performed for efficacy and safety outcomes resulting in two sets of factor rankings. After considering data availability, the final ranking included 12 clinical factors that were considered for analysis.

### Statistical analysis

As no common comparator was available, an unanchored MAIC was conducted to determine the relative efficacy and safety of liso-cel versus mosunetuzumab. Given that the eligibility criteria were largely aligned, all patients in the ITT set from TRANSCEND FL (*n* = 114) were used to compare efficacy outcomes and the treated set from TRANSCEND FL (*n* = 107) was used to compare safety outcomes.

To perform the MAIC, separate analyses were carried out sequentially for the 12 ranked clinical prognostic factors and treatment-effect modifiers. Each analysis adjusted for one additional variable at a time, following the order of ranked importance. The performance of each MAIC model was assessed based on specific criteria, including (i) effective sample size (ESS) available after adjustment, (ii) distribution of patient weights to avoid extreme patient weights, (iii) summary statistics, including SMD, to evaluate the balance between study populations, and (iv) assumption of proportional hazards for DOR and PFS.

The primary analysis aimed to balance these criteria, prioritizing ESS while mitigating extreme patient weights and adjusting for the most critical factors. In contrast, sensitivity analyses focused on adjusting for a broader set of factors for efficacy at the expense of ESS. The method-of-moments propensity score estimator was used to estimate and assign weights to patients from the TRANSCEND FL dataset [31], while ensuring an exact balancing of the clinical factors that were included in each analysis [32]. The pseudo-IPD for PFS and DOR were generated by digitizing the Kaplan-Meier survival curves from published sources and employing the Guyot et al. 2012 methodology [33].

We computed the relative efficacy of liso-cel compared with mosunetuzumab for the binary endpoints, ORR and CR rate, by estimating odds ratios (OR) and their corresponding 95% confidence intervals (CI) through weighted generalized linear models. For the time-to-event endpoints, we computed DOR and PFS hazard ratios (HR) and their respective 95% CIs using a weighted Cox proportional hazards model. The validity of the proportional hazard assumption for DOR and PFS was evaluated using the Grambsch-Therneau test, where *P <* 0.05 was considered indicative of a violation of the assumption. All statistical analyses were performed using R software, version 4.2.2 (R Core Team, Vienna, Austria; https://www.r-project.org/), based on the code outlined in the National Institute for Health and Care Excellence Evidence Synthesis Technical Support Documents Series.

## Results

### Baseline characteristics before and after matching and adjusting

Examination of baseline characteristics identified several notable differences between the TRANSCEND FL and GO29781 studies (Table 2). Moderate to large differences (SMDs ≥ 0.1) were observed in six baseline characteristics (the number of prior systemic LOTs, the occurrence of POD24, the presence of bulky disease at screening, FL International Prognostic Index (FLIPI) risk factors, double-refractory status, and history of autologous HSCT).

**Table 2:** Baseline characteristics before and after adjustment (efficacy endpoints)

	Before adjusting			After adjusting		
Baseline characteristics^a^	GO29781 enrolled (*N* = 90)	TRANSCEND FL leukapheresis set (ITT) (*N* = 114)	SMD	GO29781 enrolled (*N* = 90)	TRANSCEND FL leukapheresis set (ITT) (ESS = 71.4)	SMD
Number of prior systemic LOTs, *n* (%)^b^			0.122			0.000
2	34 (37.8)	49 (43.0)		34 (37.8)	34.1 (37.8)	
3	28 (31.1)	30 (26.3)		28 (31.1)	28.1 (31.1)	
> 3	28 (31.1)	35 (30.7)		28 (31.1)	28.1 (31.1)	
POD24, *n* (%)	47 (52.2)	53 (46.5)	0.115	47 (52.2)	47.2 (52.2)	0.000
Bulky disease at screening^c^, *n* (%)	31 (34.4)	32 (28.1)	0.138	31 (34.4)	31.1 (34.4)	0.000
FLIPI risk factor, *n* (%)			0.450			0.000
0‒1	26 (28.9)	13 (11.4)		26 (28.9)	26.1 (28.9)	
2	24 (26.7)	35 (30.7)		24 (26.7)	24.1 (26.7)	
3‒5	40 (44.4)	66 (57.9)		40 (44.4)	40.1 (44.4)	
Refractory to last therapy^d^, *n* (%)			0.044			0.000
No	28 (31.1)	32 (28.1)		28 (31.1)	28.1 (31.1)	
Yes	62 (68.9)	78 (68.4)		62 (68.9)	62.2 (68.9)	
Missing	0	4 (3.5)		0	0	
Double refractory, *n* (%)	48 (53.3)	74 (64.9)	0.237	48 (53.3)	48.2 (53.3)	0.000
ECOG PS at screening, *n* (%)			0.015			0.000
0	53 (58.9)	68 (59.6)		53 (58.9)	53.2 (58.9)	
1	37 (41.1)	46 (40.4)		37 (41.1)	37.1 (41.1)	
Sex – female, *n* (%)	35 (38.9)	42 (36.8)	0.042	35 (38.9)	35.1 (38.9)	0.000
Prior autologous HSCT, *n* (%)	19 (21.1)	34 (29.8)	0.201	19 (21.1)	19.1 (21.1)	0.000

^a^Additional baseline characteristics were considered important but unavailable for adjustment or too costly in ESS. ^b^Both GO29781 and the 3L+ cohort of TRANSCEND FL required all patients to have been exposed to ≥ 2 prior lines of therapy, including an anti-CD20 and an alkylator therapy (Additional file 1: Results S1). ^c^Bulky disease for TRANSCEND FL was aligned with the definition from GO29781, where any mass > 6 cm was considered bulky disease. ^d^Refractory to last therapy for TRANSCEND FL was aligned with the definition from GO29781, where refractory was defined as the best response to this therapy is not complete response or partial response or if the patient was assessed with progressive disease ≤ 6 months after the last treatment date

*3L+* third line or later, *CAR* chimeric antigen receptor, *ECOG PS* Eastern Cooperative Oncology Group performance status, *ESS* effective sample size, *FL* follicular lymphoma, *FLIPI* Follicular Lymphoma International Prognostic Index, *HSCT* hematopoietic stem cell transplantation, *ITT* intention to treat, *LOT* line of therapy, *POD24* progression of disease ≤ 24 months, *SMD* standardized mean difference

In the primary analysis, adjustment of patients from TRANSCEND FL substantially reduced differences between populations. This adjustment resulted in well-balanced patient characteristics between the two studies, with SMD < 0.1 for nine key baseline characteristics (Table 2). In the primary analysis for efficacy, we adjusted for LOT, POD24, bulky disease at screening, FLIPI risk factor, refractory to last therapy, double refractory to an anti-CD20 antibody and alkylating agent, Eastern Cooperative Oncology Group performance status (ECOG PS) at screening, sex, and prior receipt of autologous HSCT. Primary analyses for safety were adjusted for seven baseline characteristics, including age, bulky disease at screening, refractory to last therapy, number of prior systemic LOTs, ECOG PS at screening, Ann Arbor stage, and sex. In the primary analyses, TRANSCEND FL retained an adequate ESS of 71.4 and 81.9 for efficacy and safety comparisons, respectively. Table 2 presents a summary of the baseline characteristics in the primary analysis for efficacy endpoints both before and after adjustment. Baseline characteristics before and after adjustment for safety endpoints are presented in Additional file 1: Table S3.

### Efficacy outcomes

#### Response rates

**Fig. 1:**
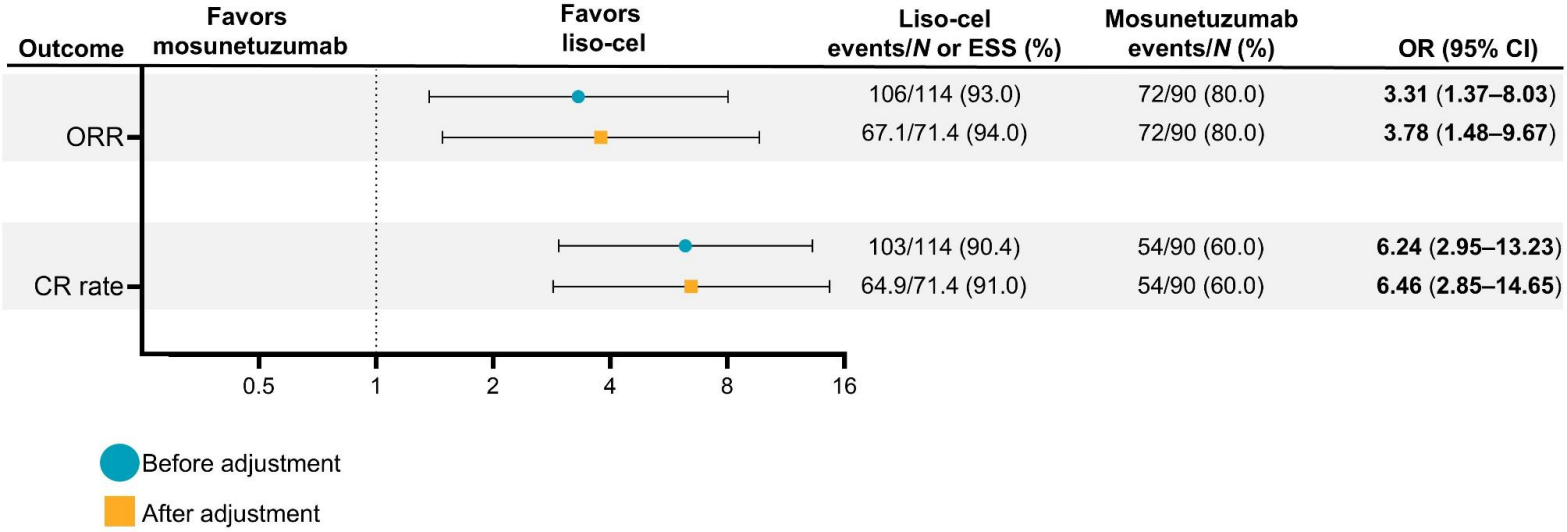
Relative treatment-effect estimates for response rates before and after adjustment. OR (95% CI) values in bold denote statistical significance. Data are in the leukapheresis efficacy set (ITT; *N* = 114). *CI* confidence interval, *CR* complete response, *ESS* effective sample size, *liso-cel* lisocabtagene maraleucel, *OR* odds ratio, *ORR* objective response rate

Overall, MAIC results showed higher response outcomes (ORR and CR rate) for liso-cel compared with mosunetuzumab (Fig. 1). Before adjustment, 93% of patients who received liso-cel responded, compared with 80% of patients who received mosunetuzumab (OR = 3.31, 95% CI 1.37‒8.03). After adjustment, the ORR for patients who received liso-cel increased to 94% (OR = 3.78, 95% CI 1.48‒9.67).

Likewise, before adjustment, 90.4% of patients who received liso-cel achieved CR, compared with 60.0% of patients who received mosunetuzumab (OR = 6.24, 95% CI 2.95‒13.23). After adjustment, the CR rate for patients treated with liso-cel increased to 91.0% versus mosunetuzumab (OR = 6.46, 95% CI 2.85‒14.65). The estimated ORR and CR rate outcomes remained consistent using the treated efficacy set for TRANSCEND FL (Additional file 1: Fig. S1).

#### Survival outcomes (DOR and PFS)

**Fig. 2:**
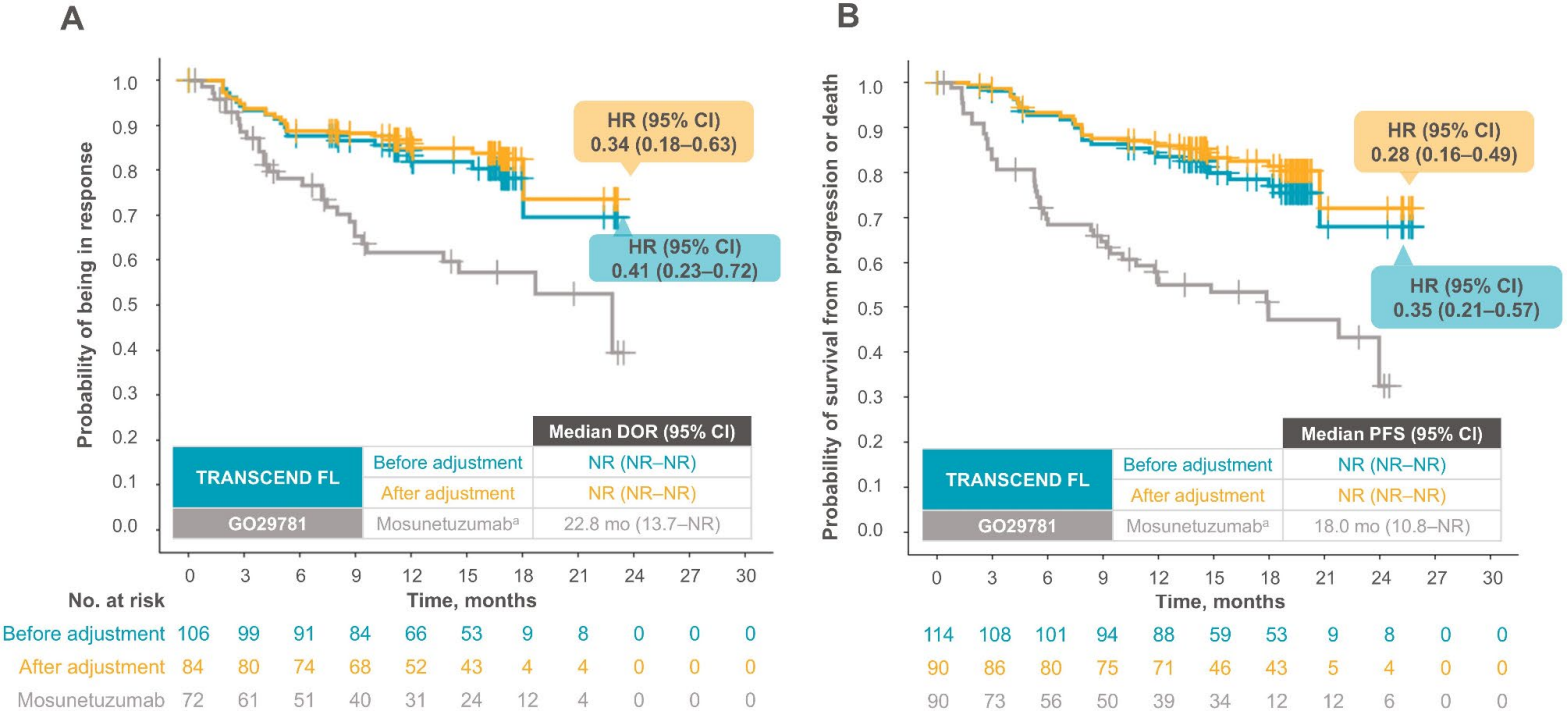
Comparison of (**A**) DOR and (**B**) PFS before and after adjustment per IRC. Data are in the leukapheresis efficacy set (ITT; *N* = 114). ^a^Data for mosunetuzumab are based on reconstructed IPD from digitizing Budde et al. 2022, Fig. 3, derived using the Guyot method [33]. *CI* confidence interval, *DOR* duration of response, *FL* follicular lymphoma, *HR* hazard ratio, *IRC* independent review committee, *ITT* intention to treat, *NR* not reached, *PFS* progression-free survival

MAIC results showed greater improvements in both DOR and PFS for liso-cel compared with mosunetuzumab before and after adjustment (Fig. 2). In the unadjusted comparison of DOR, among patients who responded to treatment (i.e., an occurrence of CR or PR), liso-cel had a lower hazard of disease progression or death (HR = 0.41, 95% CI 0.23‒0.72) compared with mosunetuzumab. Liso-cel did not reach the median DOR, while the median DOR for mosunetuzumab was 22.8 months (95% CI 13.7‒NR). After adjustment, the rate of disease progression or death among responders remained lower for liso-cel compared with mosunetuzumab (HR = 0.34, 95% CI 0.18‒0.63).

Similarly, in the unadjusted comparison of PFS, liso-cel had a lower rate of disease progression or death (HR = 0.35, 95% CI 0.21‒0.57) compared with mosunetuzumab (Fig. 2). Liso-cel did not reach the median PFS during follow-up, while the median PFS for mosunetuzumab was 18 months (95% CI 10.8‒NR). After adjustment, the rate of disease progression remained lower for liso-cel than mosunetuzumab, with a slightly reduced HR (HR = 0.28, 95% CI 0.16‒0.49). The outcomes of DOR and PFS remained consistent using the treated efficacy set for TRANSCEND FL (Additional file 1: Fig. S2).

#### Safety outcomes

**Fig. 3:**
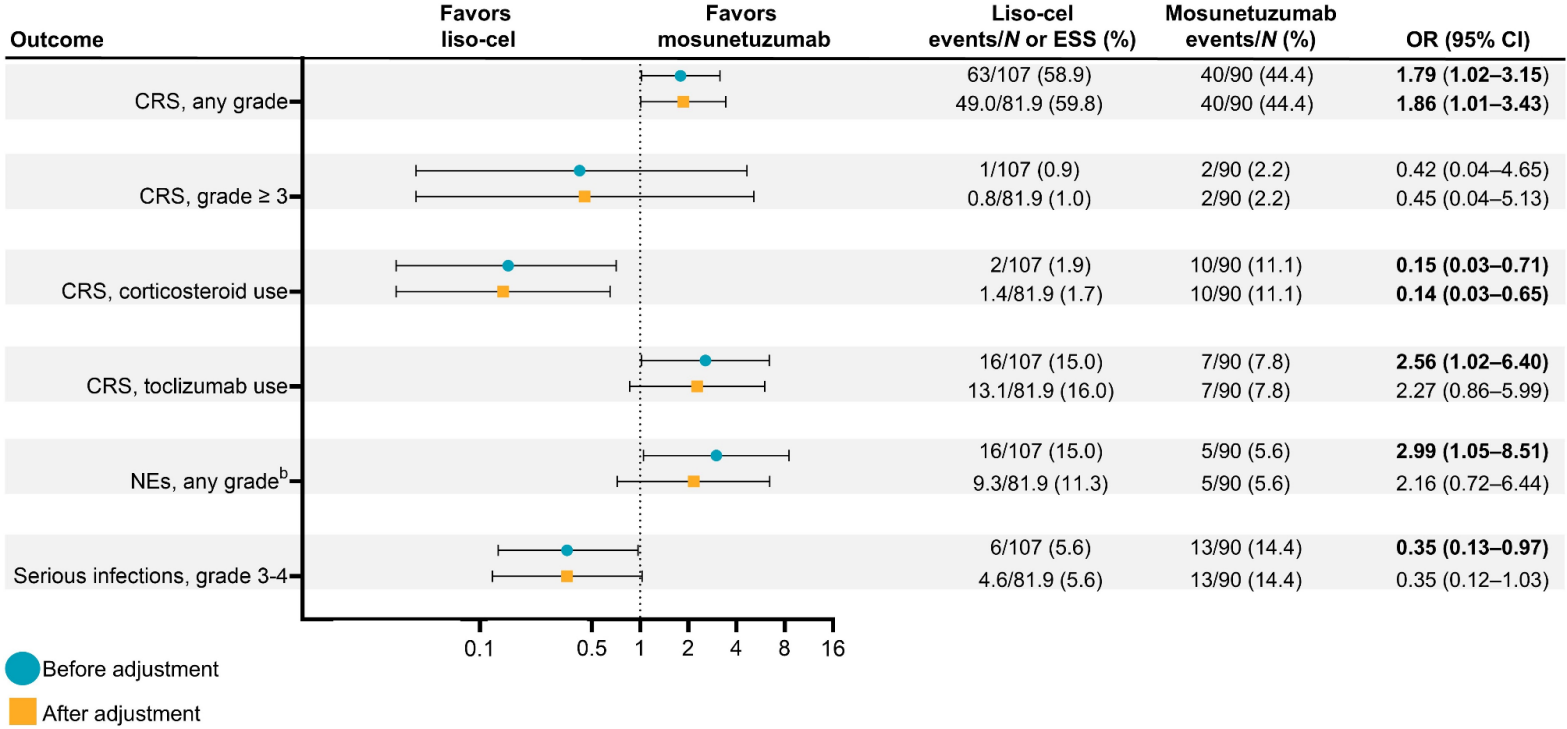
Relative treatment-effect estimates for safety outcomes before and after adjustment^a^. OR (95% CI) values in bold denote statistical significance. ^a^Safety comparisons were confounded by preventative use of corticosteroids in all patients in GO29781. ^b^NEs were defined as investigator-identified AEs related to liso-cel and graded using the National Cancer Institute Common Terminology Criteria for Adverse Events, version 5.0, per TRANSCEND FL protocol; for mosunetuzumab, NEs were defined as AEs observed by investigator assessment as related to mosunetuzumab and consistent with immune effector cell–associated neurotoxicity syndrome, including confusional state, disturbance in attention, and cognitive disorder. Note: grade ≥ 3 NEs were not compared because of limited data. *AE* adverse event, *CI* confidence interval, *CRS* cytokine release syndrome, *ESS* effective sample size, *FL* follicular lymphoma, *liso-cel* lisocabtagene maraleucel, *NE* neurological event, *OR* odds ratio

In this MAIC, safety analyses were conducted using the treated sets for the two studies, TRANSCEND FL (*N* = 107) and GO29781 (*N* = 90). Overall, most safety outcomes between patients treated with liso-cel and those treated with mosunetuzumab were found to be similar, both before and after conducting the adjustment using the MAIC method (Fig. 3). In both studies, certain grade ≥ 3 AEs occurred infrequently (grade ≥ 3 CRS: *n* = 1 in TRANSCEND FL and *n* = 2 in GO29781).

Before adjustment, liso-cel was associated with an increased incidence of any-grade CRS and a reduced incidence of grade ≥ 3 CRS compared with mosunetuzumab, which occurred infrequently in both studies (*n* = 1 in TRANSCEND FL; *n* = 2 in GO29781). For medication management of CRS, liso-cel was associated with a decreased use of corticosteroid and an increased use of tocilizumab. Liso-cel was also associated with an increased incidence of any-grade NEs and a reduced incidence of grade 3‒4 serious infections. Similarly, after adjustment, liso-cel was associated with higher incidences of any-grade CRS (OR = 1.86, 95% CI 1.01‒3.43) and had a lower incidence of grade ≥ 3 CRS (OR = 0.45, 95% CI 0.04‒5.13) compared with mosunetuzumab. For medication management of CRS, liso-cel was also associated with a reduction in the use of corticosteroids for CRS (OR = 0.14, 95% CI 0.03‒0.65), while the use of tocilizumab remained higher among patients treated with liso-cel (OR = 2.27, 95% CI 0.86‒5.99). Liso-cel was also associated with higher incidences of any-grade NEs (OR = 2.16, 95% CI 0.72‒6.44) and lower incidences of grade 3‒4 serious infections (OR = 0.35, 95% CI 0.12‒1.03) compared with mosunetuzumab.

## Discussion

In the absence of a prospective, randomized comparison, results from this MAIC of liso-cel with mosunetuzumab demonstrate better efficacy outcomes for patients with 3L+ R/R FL treated with liso-cel, including improvements in ORR, CR rate, PFS, and DOR. Safety outcomes further support treatment with liso-cel with reduced incidence of grade ≥ 3 CRS and grade 3‒4 serious infections; however, given the low incidence of safety events, specifically grade ≥ 3 CRS events in both studies, the safety findings need to be validated by real-world data. To our knowledge, this is the first study to provide comparative data supporting the use of liso-cel in 3L+ R/R FL.

Our findings are consistent with recently published ITCs between the CAR T-cell therapies tisa-cel and axi-cel versus mosunetuzumab [23, 24, 34]. In an MAIC comparing tisa-cel versus mosunetuzumab using data from ELARA and GO29781, respectively, tisa-cel demonstrated favorable efficacy outcomes after adjusting for differences in patient characteristics [23]. In particular, ORR was 11% higher for tisa-cel compared with mosunetuzumab (91% vs. 80%, *P* < 0.05), and results were similar for CR rate [23]. Tisa-cel was also reported to have a lower rate of disease progression or death compared with mosunetuzumab (HR = 0.54, 95% CI 0.54‒0.90, *P* < 0.05) [23]. Most safety outcomes were found to be comparable between the two treatments [23]. Another MAIC comparing axi-cel versus mosunetuzumab using data from ZUMA-5 and GO29781 also demonstrated that CAR T-cell therapy had better efficacy compared with mosunetuzumab [24]. Axi-cel was associated with a higher ORR and CR rate (OR = 4.74, 95% CI 1.73‒12.97; OR = 3.67, 95% CI 1.88‒7.18, respectively), in addition to improved PFS (HR = 0.38, 95% CI 0.23‒0.61) and DOR (HR = 0.45, 95% CI 0.26‒0.77) [24]. Safety outcomes were comparable when restricted to only grade 3 or 4 treatment-related AEs [24]. However, across all grades, patients treated with axi-cel experienced CRS and NEs at a greater rate versus mosunetuzumab [24]. A third MAIC, which compared mosunetuzumab (using data from GO29781) against a variety of therapies from multiple pharmacologic classes, including CAR T-cell therapy (using data from ZUMA-5 and ELARA), showed results favoring axi-cel for the outcomes of ORR (OR = 0.300, 95% CI 0.147‒1.212), CR rate (OR = 0.336, 95% CI 0.179‒0.635), and PFS (HR = 2.205, 95% CI 1.339‒3.244) and MAIC results were similar between mosunetuzumab versus tisa-cel for ORR (OR = 0.641, 95% CI 0.306‒1.661), CR rate (OR = 0.619, 95% CI 0.298‒1.268), PFS (HR = 1.784, 95% CI 1.032‒2.629), and OS (HR = 1.049, 95% CI 0.157‒2.458) [34]. Specific safety outcomes were not compared in this MAIC.

This study benefits from several key strengths that enhance its validity and reliability. The consistency of results across multiple ITCs between CAR T-cell therapies versus mosunetuzumab confirms the robustness of the reported efficacy outcomes in the current study and highlights the favorable efficacy of CAR T-cell therapies in general for patients with R/R FL. Another notable strength of the current analysis was that a rigorous, multifaceted approach was undertaken to identify and rank-order clinically relevant factors and treatment-effect modifiers. During this process, a panel of clinical experts was convened to rank-order clinical factors based on prognostic strength and degree of effect modification. These were compiled to generate a final rank-ordered list of evidence-informed factors.

Unanchored ITCs are susceptible to various sources of bias, an inherent limitation of this type of analysis. Although rigorous efforts were made to mitigate bias in this study, not all factors could be adjusted for due to lack of reporting. Nevertheless, the rigorous variable selection process ensured that the most important factors were adjusted. OS was not analyzed because of low event rate (i.e., 13 out of 114 patients in TRANSCEND FL and 8 out of 90 patients in GO29781) and limited follow-up from both studies. Median survival was also NR in both studies with 12-month survival at 92.1% in TRANSCEND FL and 93.0% in GO29781. MAIC estimates using the current data from both studies may be unreliable with wide CIs that are not meaningful. Given the indolent nature of FL, data with longer follow-up (i.e., clinical trial data or real-world evidence) is needed to evaluate differences in OS. A further limitation of this study is the potential for the comparison of safety endpoints to be confounded by preventative corticosteroid use in GO29781, differences in endpoint definitions between studies (e.g., NEs), and the differences in reporting windows for safety events. In the GO29781 study, treatment-emergent AEs were reported until 90 days after the last administration of mosunetuzumab or until the study’s completion, resulting in a reporting window that is much longer than the 90-day reporting window after infusion in TRANSCEND FL. However, this discrepancy had minimal impact on the safety outcomes, and it is likely that the majority of treatment-emergent AEs occur within 90 days after administration. Despite these limitations, both the primary and sensitivity analyses showed congruency and demonstrated that liso-cel was associated with better efficacy outcomes compared with mosunetuzumab.

Results from the current analysis highlight a potentially favorable benefit–risk profile of liso-cel over mosunetuzumab in the 3L+ R/R FL population. These results may carry significant implications for clinical practice, potentially influencing the establishment of a SOC for FL in the advanced stage (i.e., 3L+) as well as a potential for a shift towards increased outpatient administration. A recent retrospective study that compared inpatient versus outpatient monitoring after liso-cel infusion in patients with 3L+ R/R diffuse large B-cell lymphoma found that the outpatients had lower overall health care resource utilization, resulting in a mean 6-month cost savings of $53,833 compared with inpatient monitoring [35, 36]. Mosunetuzumab can also be administered in an outpatient setting, potentially making it a suitable option for frailer patients or those with limited access to treatment centers that administer CAR T-cell therapy. Overall, the transition of patient care to outpatient monitoring is a positive development for patients with 3L+ R/R FL that could lead to a decrease in hospital utilization (e.g., inpatient and intensive care unit stays).

Given the limited options for treatment of 3L+ R/R FL, head-to-head clinical trials and real-world evidence comparing the safety and efficacy of liso-cel (and other CAR T-cell therapies) with mosunetuzumab would provide valuable insights to help inform clinical decisions in a disease space that has historically been limited in treatment options.

## Conclusions

In summary, unanchored MAICs using IPD from TRANSCEND FL and published data from GO29781 were used to derive ITCs while accounting for between-study differences. After adjusting for important clinical prognostic and treatment-effect modifiers, liso-cel was found to offer a favorable benefit–risk profile relative to mosunetuzumab as a 3L+ treatment for R/R FL.

## Electronic supplementary material

Below is the link to the electronic supplementary material.

Supplementary Material 1

## Data Availability

Bristol Myers Squibb policy on data sharing may be found at https://www.bms.com/researchers-and-partners/independent-research/data-sharing-request-process.html.

## Abbreviations

2L: Second line
3L+: Third line or later
AE: Adverse event
Axi-cel: Axicabtagene ciloleucel
CAR: Chimeric antigen receptor
CI: Confidence interval
CR: Complete response
CRS: Cytokine release syndrome
DOR: Duration of response
ECOG PS: Eastern Cooperative Oncology Group performance status
ESS: Effective sample size
FDA: United States Food and Drug Administration
FL: Follicular lymphoma
FLIPI: Follicular Lymphoma International Prognostic Index
HR: Hazard ratio
HSCT: Hematopoietic stem cell transplantation
IPD: Individual patient data
IQR: Interquartile range
IRC: Independent review committee
ITC: Indirect treatment comparison
ITT: Intention to treat
Liso-cel: Lisocabtagene maraleucel
LOT: Line of therapy
MAIC: Matching-adjusted indirect comparison
NE: Neurological event
NHL: Non-Hodgkin lymphoma
NR: Not reached
OR: Odds ratio
ORR: Objective response rate
OS: Overall survival
PFS: Progression-free survival
POD24: Progression of disease ≤ 24 months
PR: Partial response
R/R: Relapsed or refractory
SMD: Standardized mean difference
SOC: Standard of care
Tisa-cel: Tisagenlecleucel
