# Supplementary material for: Matching-adjusted indirect comparison of efficacy and safety of lisocabtagene maraleucel and mosunetuzumab for the treatment of third-line or later relapsed or refractory follicular lymphoma
Source: Exp Hematol Oncol. 2025 Mar 5;14:30. doi: 10.1186/s40164-025-00610-1 (PMC11881270; doi:10.1186/s40164-025-00610-1)
Supplement: Supplementary file 1 — Supplementary Material 1 [file 40164_2025_610_MOESM1_ESM.docx]

**Additional file**

**Matching-adjusted indirect comparison of efficacy and safety of lisocabtagene maraleucel and mosunetuzumab for the treatment of third-line or later relapsed or refractory follicular lymphoma**

Loretta J. Nastoupil, Ashley Bonner, Pearl Wang, et al.

**Table of contents**

[Table S1 Definitions for baseline characteristics in TRANSCEND FL and GO29781 and actions taken 3](#_Toc187145930)

[Table S2 Factor-ranking outcomes 5](#_Toc187145931)

[Table S3 Baseline characteristics before and after adjustment (safety outcomes) 7](#_Toc187145932)

[Results. S1 Prior lines of therapy in GO29781 and TRANSCEND FL 8](#_Toc187145933)

[Fig. S1 Relative treatment-effect estimates for response rates before and after adjustment^a^ 9](#_Toc187145934)

[Fig. S2 Comparison of (A) DOR and (B) PFS before and after adjustment per IRC 10](#_Toc187145935)

[References 11](#_Toc187145936)

# Table S1 Definitions for baseline characteristics in TRANSCEND FL and GO29781 and actions taken

| **Baseline characteristics** | **TRANSCEND FL  (liso-cel)** | **GO29781 (mosunetuzumab)** | **Action taken in TRANSCEND FL IPD and rationale** |
| --- | --- | --- | --- |
| Age | In years | In years | None |
| Bulky disease at screening | Criterion 1: Single mass ≥ 7 cm OR  Criterion 2: ≥ 3 nodal sites with each side having a node that is > 3 cm | > 6 cm at study entry | Criterion 1 was redefined in TRANSCEND FL to a threshold of > 6 cm for single mass and criterion 2 was removed |
| Bridging therapy | Allowed | Not applicable | None |
| Corticosteroid  premedication | Not applied | Part of the treatment regimen in cycle 1 and 2 | None |
| Disease stage | I, II, III, IV | I, II, III, IV | None |
| ECOG PS at screening | 0, 1 | 0, 1 | None |
| Number of prior LOTs | At least one line must be a combination, which included anti-CD20 (e.g., rituximab, obinutuzumab) and an alkylating agent | At least two prior lines of systemic therapy with an anti-CD20–directed therapy and an alkylating agent | Redefined in TRANSCEND FL to align with GO29781 by removing monotherapy rituximab and obinutuzumab |
| POD24 | Progression of disease ≤ 24 months from the start of initial therapy | Progression of disease  ≤ 24 months from the start of initial therapy | None |
| Prior allogenic HSCT | Not allowed (allogenic HSCT within 90 days of leukapheresis is excluded) | Not allowed | None |
| Prior autologous HSCT | Allowed | Allowed (autologous HSCT within 100 days of first mosunetuzumab administration is excluded) | None |
| Secondary CNS involvement at time of treatment | Allowed | Not allowed (excluded per protocol) | None |
| Sex | Male; female | Male; female | None |
| R/R to last therapy | Refractory: best response to last therapy as SD or PD after prior therapy  Relapse: no criteria | Refractory: defined as refractory if the best response to this therapy is not CR or PR  Relapse: if the patient was assessed with PD within 6 months after the last treatment date of this therapy | Criterion 2 was redefined in TRANSCEND FL to align with GO29781 |

*CNS* central nervous system, *CR* complete response, *ECOG PS* Eastern Cooperative Oncology Group performance status, *FL* follicular lymphoma, *HSCT* hematopoietic stem cell transplantation, *IPD* individual patient data, *liso-cel* lisocabtagene maraleucel, *LOT* line of therapy, *PD* progressive disease, *POD24* progression of disease ≤ 24 months, *PR* partial response, *R/R* relapsed or refractory, *SD* stable disease

# Table S2 Factor-ranking outcomes

| **Factor name** | **Factor-rank efficacy** | **Factor-rank safety** | **Reported by GO29781** | **Factors used in primary analysis** | | **Factors used in sensitivity analysis^a^** |
| --- | --- | --- | --- | --- | --- | --- |
|  |  |  |  | **Tisa-cel vs. mosunetuzumab [1]** | **Axi-cel vs. mosunetuzumab [2]** | **Axi-cel vs. mosunetuzumab [2]** |
| Number of prior LOTs | 1 | 6 | Yes | Used | Used | Used |
| POD24 from initiating first anti-CD20 mAb-containing therapy | 2 |  | Yes | Used | Used | Used |
| Bulky disease/high tumor bulk | 3 | 3 | Yes |  | Used | Used |
| FLIPI score | 4 |  | Yes | Used | Used | Used |
| Refractory to last previous therapy | 5 | 5 | Yes | Used | Used | Used |
| Disease stage | 6 | 10 | Yes | Used | Used | Used |
| Age | 7 | 1 | Yes | Used | Used | Used |
| Race | 8 | 12 | Yes | Used |  | Used |
| Double-refractory status | 9 |  | Yes | Used | Used | Used |
| Sum of the product of perpendicular diameters, mm^2^ | 10 |  | No |  |  |  |
| Modified GELF criteria met at time of most recent relapse | 11 |  | No |  |  |  |
| Number of nodal sites | 12 | 9 | No |  |  |  |
| Time from last treatment | 13 | 4 | No |  |  |  |
| Lymphoma present in the bone marrow as assessed by bone marrow biopsy | 14 |  | No |  |  |  |
| LDH test (upper limit of normal) | 15 | 2 | No |  |  |  |
| Size of largest nodal mass, cm | 16 | 7 | No |  |  |  |
| ECOG PS score | 17 | 8 | Yes | Used | Used | Used |
| Histological subtypes | 18 |  | No |  |  |  |
| Relapsed or refractory status | 19 |  | No |  |  |  |
| Sex | 20 | 11 | Yes | Used |  | Used |
| Previous autologous HSCT | 21 |  | Yes | Used | Used | Used |

^a^Fowler, Schuster [1] did not report a sensitivity analysis.

*Axi-cel* axicabtagene ciloleucel, *ECOG PS* Eastern Cooperative Oncology Group performance status, *FLIPI* Follicular Lymphoma International Prognostic Index, *GELF* Groupe d'Etude des Lymphomes Folliculaires, *HSCT* hematopoietic stem cell transplantation, *LDH* lactate dehydrogenase, *LOT* line of therapy, *mAb* monoclonal antibody, *POD24* progression of disease ≤ 24 months, *tisa-cel* tisagenlecleucel

# Table S3 Baseline characteristics before and after adjustment (safety outcomes)

|  | **Before adjusting** | | | **After adjusting** | | |
| --- | --- | --- | --- | --- | --- | --- |
| **Baseline characteristics^a^** | **GO29781**  **enrolled**  **(N = 90)** | **TRANSCEND FL**  **treated set**  **(N = 107)** | **SMD** | **GO29781**  **enrolled**  **(N = 90)** | **TRANSCEND FL**  **treated set**  **(ESS = 81.9)** | **SMD** |
| Number of prior systemic LOTs,  n (%)  2  3  > 3 | 34 (37.8)  28 (31.1)  28 (31.1) | 48 (44.9)  26 (24.3)  33 (30.8) | 0.171 | 34 (37.8)  28 (31.1)  28 (31.1) | 34.7 (37.8)  28.6 (31.1)  28.6 (31.1) | 0.000 |
| Age, mean (SD) | 60 (10.4) | 61.3 (10.3) | 0.121 | 60 (10.4) | 60 (10.4) | 0.000 |
| Bulky disease at screening^b^, n (%) | 31 (34.4) | 29.0 (27.1) | 0.16 | 31 (34.4) | 31.7 (34.4) | 0.000 |
| Refractory to last therapy^c^, n (%)  No  Yes  Missing | 28 (31.1)  62 (68.9)  0 | 31 (29.0)  72 (67.3)  4 (3.7) | 0.022 | 28 (31.1)  62 (68.9)  0 | 28.6 (31.1)  63.3 (68.9)  0 | 0.000 |
| Ann Arbor stage (grouped), n (%)  I or II  III or IV | 21 (23.3)  69 (76.7) | 12 (11.2)  95 (88.8) | 0.325 | 21 (23.3)  69 (76.7) | 21.4 (23.3)  70.4 (76.7) | 0.000 |
| ECOG PS at screening, n (%)  0  1 | 53 (58.9)  37 (41.1) | 65 (60.7)  42 (39.3) | 0.038 | 53 (58.9)  37 (41.1) | 54.1 (58.9)  37.8 (41.1) | 0.000 |
| Sex – female,  n (%) | 35 (38.9) | 41 (38.3) | 0.012 | 35 (38.9) | 35.7 (38.9) | 0.000 |

^a^Additional baseline characteristics were considered important but unavailable for adjustment or too costly in ESS. ^b^Bulky disease for TRANSCEND FL was aligned with the definition from GO29781, where any mass > 6 cm was considered bulky disease. ^c^Refractory to last therapy for TRANSCEND FL was aligned with the definition from GO29781, where refractory was defined as the best response to this therapy is not complete response or partial response or if the patient was assessed with progressive disease ≤ 6 months after the last treatment date.

*ECOG PS* Eastern Cooperative Oncology Group performance status, *ESS* effective sample size, *FL* follicular lymphoma, *LOT* line of therapy, *SD* standard deviation, *SMD* standardized mean difference

# Results. S1 Prior lines of therapy in GO29781 and TRANSCEND FL

In GO29781, other prior therapies included anthracyclines (82%), autologous stem cell transplantation (21%), phosphatidylinositol 3-kinase inhibitors (19%), immunomodulatory drugs (14%), and CAR T-cell therapy (3%).[3]

In TRANSCEND FL, other prior therapies in patients with 3L+ FL included bendamustine (61%), autologous stem cell transplantation (31%), phosphatidylinositol 3-kinase inhibitors (22%), and rituximab and lenalidomide (21%).[4]

# Fig. S1 Relative treatment-effect estimates for response rates before and after adjustment^a^


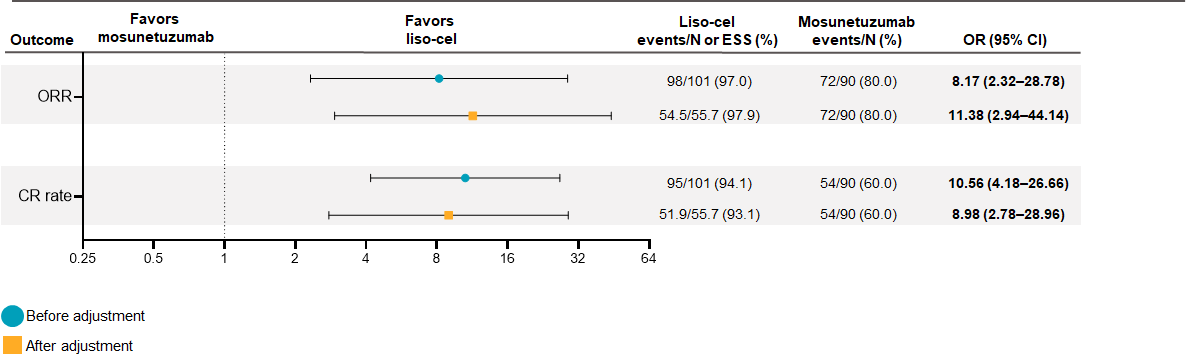


OR (95% CI) values in bold denote statistical significance. ^a^Data are in the liso-cel–treated efficacy set (N = 101). Liso-cel–treated efficacy set is based on the analysis of outcomes from the infusion of liso-cel. *CI* confidence interval, *CR* complete response, *ESS* effective sample size, *liso-cel* lisocabtagene maraleucel, *OR* odds ratio, *ORR* objective response rate

# Fig. S2 Comparison of (A) DOR and (B) PFS before and after adjustment per IRC


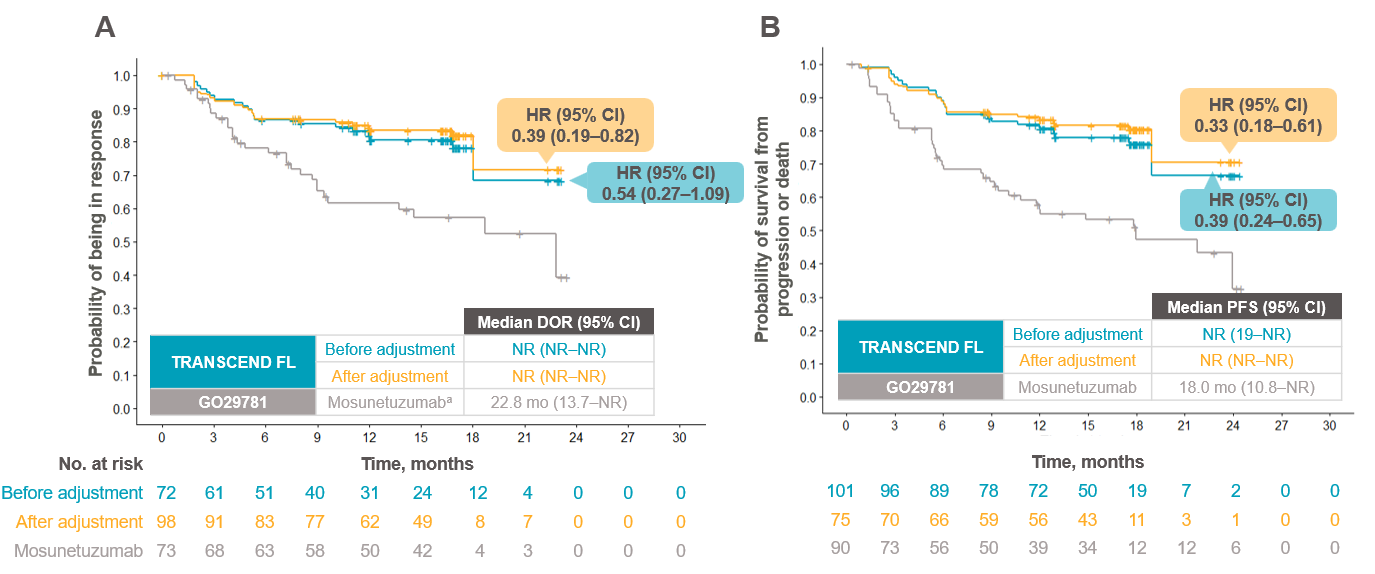


Data are in the liso-cel–treated efficacy set (N = 101). ^a^Data for mosunetuzumab are based on reconstructed IPD from digitizing Budde 2022, Figure 3A, derived using Guyot method [5]. *CI* confidence interval, *DOR* duration of response, *FL* follicular lymphoma, *HR* hazard ratio, *IRC* independent review committee, *liso‑cel* lisocabtagene maraleucel, *NR* not reached, *PFS* progression-free survival

# References

1. Fowler NH, Schuster SJ, Yang H, Xiang C, Ramos R, Maier HJ, et al. Matching-adjusted indirect comparison of efficacy and safety for tisagenlecleucel and mosunetuzumab in patients (pts) with relapsed/refractory follicular lymphoma (r/r FL). Transplant Cell Ther. 2023;29(2 suppl):S194.

2. Ray MD, Kanters S, Beygi S, Best T, Wulff J, Limbrick-Oldfield EH, et al. Matching-adjusted indirect comparisons of axi-cel to mosunetuzumab for the treatment of relapsed/refractory follicular lymphoma. Hematol Oncol. 2023;41(52):522‒33.

3. Budde LE, Sehn LH, Matasar M, Schuster SJ, Assouline S, Giri P, et al. Safety and efficacy of mosunetuzumab, a bispecific antibody, in patients with relapsed or refractory follicular lymphoma: a single-arm, multicentre, phase 2 study. Lancet Oncol. 2022;23(8):1055‒65.

4. Morschhauser F, Dahiya S, Palomba ML, Martin Garcia-Sancho A, Reguera Ortega JL, Kuruvilla J, et al. Lisocabtagene maraleucel in follicular lymphoma: the phase 2 TRANSCEND FL study. Nat Med. 2024. doi: 10.1038/s41591-024-02986-9.

5. Guyot P, Ades AE, Ouwens MJ, Welton NJ. Enhanced secondary analysis of survival data: reconstructing the data from published Kaplan-Meier survival curves. BMC Med Res Methodol. 2012;12:9.
